# Supplementary material for: Patterns of Intron Gain and Loss in Fungi
Source: PLoS Biol. 2004 Nov 30;2(12):e422. doi: 10.1371/journal.pbio.0020422 (PMC532390; doi:10.1371/journal.pbio.0020422)
Supplement: Table S1 — Also available at http://genes.mit.edu/NielsenEtAl/. (4.3 MB ZIP). [file pbio.0020422.st001.zip › NielsenEtAl/html/913.html]

AN2511.1.NCU05781.1.MG08269.1.FG02639.1


```
 CLUSTAL W (1.82) Multiple Sequence Alignments - Introns Inserted


Sequence 1: MG08269.1	236 aa
Sequence 2: FG02639.1	207 aa
Sequence 3: NCU05781.1	244 aa
Sequence 4: AN2511.1	225 aa
Alignment Length: 247 aa
Number Identitical Residues: 129 aa
Alignment Score (without introns) 5440


MG08269.1 	MADKAAE--KDKDKSKVHKLSLKG~SAKLVAEF0-KYGLNMLG~TAMNSPLRPPQAFPH~
NCU05781.1	MSSKAPEKEKDKDKSKVHRLSLKG~SAKLVAEF0FQYSIHTIL2FQRG--VYPAEDFTT~
FG02639.1 	MSSKETSKSKDKEKSKVHKLSLKG~SARLVAEF0-KYGLNML-~---------------~
AN2511.1  	MST-----SKDKSKDKVHKLSIKG1SSKLVAEF0FEYSINSIL2FQRG--VYPPEDFTT2
          	*:      .***.*.***:**:** *::*****  :*.:: :     .    ...  .  

MG08269.1 	PQNAADPRTTV~SADDQVKAYIKKIMSQLDRWMVKGKISKLVIVITDKDTGEHVERWQFD
NCU05781.1	VKKYG-LNMLV~SADDQVRAYIKRIMSQLHKWMVGSKISKLVIVITDKDTGEHVERWQFD
FG02639.1 	----------V1SADDQVKAYIKKIMSQLDKWMVGGKISKLVIVITDKDTGEHVERWQFD
AN2511.1  	VKKYG-LNMLV1TADDQVKAYIKKIMSQLNKWMLGGKISKLVVVITDKETGEHVERWQFD
          	 .. .     * :*****:****:*****.:**: .******:*****:***********

MG08269.1 	~VQIFGGSSKKSKSSSKSAASASAGQENS----SAAS~DAAQAP----EKTEAEIQAEIA
NCU05781.1	0VQIFSKAAQSKTKSQPASATTDDNQNQSPNENAAAS~TAASTPTPPLDKTEAEIQAEIA
FG02639.1 	0VQIFQP-VKKSKSSK----SAPKDQENP----AAGS~AAPTAP----EKTEPEIQAEIA
AN2511.1  	~VQIFGK--QKSQSTR-----KSGDKEND-----TPG2DVGQPE--TVEKTEKQIQEEIQ
          	 ****    :.. .:         .:::      : .  .  .   . :*** :** ** 

MG08269.1 	AIFRQITASVTFLPQLAGDCTFNVLVYADADSEVPVEWGDSDAKEIENGERVQLRGFSTA
NCU05781.1	AIFRQITASVTFLPQLSGDCTFNVLVYADADSDVPVEWGDSDAKEIVNGERVQLRGFSTT
FG02639.1 	AIFRQITASVTFLPQLNGDCTFNVLVYADADSEVPVEWGDSDAKEIENGEKVQLRGFSTA
AN2511.1  	AIFRQITASVTFLPVLDGDCTFNVLVYADADSDVPVEWGDSDAKDIKNPEKVQLRSFSTN
          	************** * ***************:***********:* * *:****.*** 

MG08269.1 	SHRVDTLVSYRLTD
NCU05781.1	NHRVDTLVSYRLAD
FG02639.1 	NHRVDTLVSYRFSD
AN2511.1  	NHRVETMVSYRLAD
          	.***:*:****::*
```
